# Blood–brain barrier permeability measured using dynamic contrast‐enhanced magnetic resonance imaging: a validation study

**DOI:** 10.1113/JP276887

**Published:** 2018-11-29

**Authors:** Aravinthan Varatharaj, Maria Liljeroth, Angela Darekar, Henrik B.W. Larsson, Ian Galea, Stig P. Cramer

**Affiliations:** ^1^ Clinical Neurosciences Clinical and Experimental Sciences Faculty of Medicine University of Southampton Southampton UK; ^2^ Department of Medical Physics University Hospital Southampton NHS Foundation Trust Southampton UK; ^3^ Functional Imaging Unit Department of Clinical Physiology Nuclear Medicine and PET, Rigshospitalet Copenhagen Denmark

**Keywords:** Blood‐brain barrier, Cerebral blood flow, Magnetic Resonance Imaging

## Abstract

**Key points:**

- The blood–brain barrier (BBB) is an important and dynamic structure which contributes to homeostasis in the central nervous system.
- BBB permeability changes occur in health and disease but measurement of BBB permeability in humans is not straightforward.
- Dynamic contrast‐enhanced magnetic resonance imaging (DCE‐MRI) can be used to model the movement of gadolinium contrast into the brain, expressed as the influx constant *K*_i_.
- Here evidence is provided that *K*_i_ as measured by DCE‐MRI behaves as expected for a marker of overall BBB leakage.
- These results support the use of DCE‐MRI for *in vivo* studies of human BBB permeability in health and disease.

**Abstract:**

Blood–brain barrier (BBB) leakage can be measured using dynamic contrast‐enhanced magnetic resonance imaging (DCE‐MRI) as the influx constant *K*_i_. To validate this method we compared measured *K*_i_ with biological expectations, namely (1) higher *K*_i_ in healthy individual grey matter (GM) *versus* white matter (WM), (2) GM/WM cerebral blood volume (CBV) ratio close to the histologically established GM/WM vascular density ratio, (3) higher *K*_i_ in visibly enhancing multiple sclerosis (MS) lesions *versus* MS normal appearing white matter (NAWM), and (4) higher *K*_i_ in MS NAWM *versus* healthy individual NAWM. We recruited 13 healthy individuals and 12 patients with MS and performed whole‐brain 3D DCE‐MRI at 3 T. *K*_i_ and CBV were calculated using Patlak modelling for manual regions of interest (ROI) and segmented tissue masks. *K*_i_ was higher in control GM *versus* WM (*P* = 0.001). CBV was higher in GM *versus* WM (*P* = 0.005, mean ratio 1.9). *K*_i_ was higher in visibly enhancing MS lesions *versus* MS NAWM (*P* = 0.002), and in MS NAWM *versus* controls (*P* = 0.014). Bland–Altman analysis showed no significant difference between ROI and segmentation methods (*P* = 0.638) and an intra‐class correlation coefficient showed moderate single measure consistency (0.610). *K*_i_ behaves as expected for a compound marker of permeability and surface area. The GM/WM CBV ratio measured by this technique is in agreement with the literature. This adds evidence to the validity of *K*_i_ measured by DCE‐MRI as a marker of overall BBB leakage.

## Key points

- The blood–brain barrier (BBB) is an important and dynamic structure which contributes to homeostasis in the central nervous system.
- BBB permeability changes occur in health and disease but measurement of BBB permeability in humans is not straightforward.
- Dynamic contrast‐enhanced magnetic resonance imaging (DCE‐MRI) can be used to model the movement of gadolinium contrast into the brain, expressed as the influx constant *K*_i_.
- Here evidence is provided that *K*_i_ as measured by DCE‐MRI behaves as expected for a marker of overall BBB leakage.
- These results support the use of DCE‐MRI for *in vivo* studies of human BBB permeability in health and disease.

## Introduction

The blood–brain barrier (BBB) is important for the maintenance of a stable microenvironment in the central nervous system (CNS), and in the regulation of solute and cellular traffic between systemic and CNS compartments (Abbott *et al*. 2010). BBB permeability is a physiological phenomenon, present in the healthy state; however, it increases with age and in disease (Elwood *et al*. 2017). Measuring BBB permeability in humans is not straightforward. The cerebrospinal fluid/serum albumin ratio is a common and well‐established method to assess BBB permeability, but it is invasive and there are concerns that it does not reliably reflect BBB permeability, with a substantial influence from CSF flow (Reiber, 1994). Neuro‐imaging after an intravenous injection of tracer is an attractive technique to measure BBB permeability, and while positron emission tomography was first utilized in this way (Iannotti *et al*. 1987), there are disadvantages which have precluded its widespread use, including radioactivity and suboptimal resolution. Dynamic contrast‐enhanced magnetic resonance imaging (DCE‐MRI) is a newer technique which can measure all levels of BBB leakiness, including levels invisible to conventional imaging (Cramer *et al*. 2014). To achieve this high sensitivity, the brain is scanned continuously for a period of time (e.g. 15 min) to acquire kinetic data capturing the periods before, during and after contrast injection. Mathematical modelling is then used to derive an index of BBB leakiness (the transfer coefficient, *K*_i_), using values derived from regions of interest (ROIs) representing the concentration in the internal carotid artery and in the brain parenchyma itself. *K*_i_ is expressed as a rate constant per gram of brain tissue (ml (100 g)^−1^ min^−1^); it is the permeability‐surface area (*PS*) product including the effect of regional cerebral blood flow (CBF). The *PS* product adjusted for regional vascular surface area (*S*) is the permeability (*P*) in cm min^−1^. Hence one would expect a higher *K*_i_ in areas with a higher vascular surface area available for tracer exchange. The latter phenomenon can be utilized as one of several ways to validate *K*_i_ derived from DCE‐MRI in healthy control individuals, since it has been histologically established that vascular density in the grey matter (GM) is higher than in white matter (WM) (Lierse & Horstmann, 1965). Assuming a very simple vascular architecture model comprising cylindrical vessels with constant radius, it can be seen that both S and CBV scale with vessel density, and CBV should then predict *K*_i_.

A neuroimaging technique measuring BBB permeability should be able to detect increased permeability in central nervous system conditions where the BBB is impaired, such as multiple sclerosis (MS). In MS, highly active inflammatory lesions have pronounced BBB leakage and therefore visibly enhance on T1‐weighted MRI after intravenous gadolinium administration. In addition, BBB abnormalities in the ‘normal‐appearing brain tissue’ have been demonstrated histologically (Kirk *et al*. 2003; Vos *et al*. 2005), and DCE‐MRI can detect this slightly higher *K*_i_ in normal appearing white matter (NAWM) (Cramer *et al*. 2014). However, a number of observations suggest caution in interpreting *K*_i_ as a BBB permeability marker. First, other studies using variations of DCE‐MRI have only shown insignificant trends for higher *K*_i_ values in MS subjects (Silver *et al*. 2001; Lund *et al*. 2013; Taheri *et al*. 2013). Secondly, we reported a correlation between *K*_i_, CSF leukocyte count, and matrix metalloprotease 9 (Cramer *et al*. 2015), an enzyme involved in leukocyte infiltration, in MS patients. Hence it is possible that the higher *K*_i_ seen in MS reflects low‐level leukocyte recruitment into the brain parenchyma, rather than BBB permeability to solutes – the movements of solutes and cells across the BBB are distinct processes (Bechmann *et al*. 2007). Thirdly, it is possible that gadolinium may reach the brain parenchyma without permeating through the BBB, namely across the choroid plexus into the CSF (Jost *et al*. 2017), and subsequent equilibration with the brain interstitial fluid via the glymphatic pathway (Iliff *et al*. 2012).

Essentially, it remains to be conclusively demonstrated that *K*_i_ as measured by DCE‐MRI is a valid marker of BBB leakage, and this is important given the technical challenges of DCE‐MRI and the potential for a false positive error. In order to validate DCE‐MRI‐derived *K*_i_ as a BBB permeability marker, we hypothesized that DCE‐MRI‐derived *K*_i_ is in line with a number of biological expectations, namely (1) higher *K*_i_ in healthy individual grey *versus* white matter, (2) grey/white matter vascular surface area ratio close to the histologically established value, (3) higher *K*_i_ in contrast‐enhancing white matter multiple sclerosis (MS) lesions *versus* MS NAWM, and (4) higher *K*_i_ in MS NAWM *versus* healthy individual NAWM. By acquiring data in three dimensions, we also aimed to test whether automated segmentation yields similar NAWM *K*_i_ results to manual region of interest analysis.

## Methods

### Ethical approval

The study was approved by the National Research Ethics Service Committee South Central (reference 12/SC/0176). Experiments were conducted in accordance with the *Declaration of Helsinki* and all subjects gave informed written consent.

### Study participants

Healthy adult individuals were recruited by advertisement; inclusion criteria were as follows: no systemic or neurological disease (including migraine), no regular medication use, and no family history of MS. Adults with relapsing–remitting MS (RRMS) were recruited from the MS service at the Wessex Neurological Centre, Southampton, UK. Demographic and clinical data was collected including: MS disease duration, Expanded Disability Status Scale (EDSS), treatment status, relapses in the prior 12 months, steroid use in the prior 12 months, concomitant medication, and other medical conditions. All subjects had normal renal function. Subjects were scanned in an interleaved fashion to prevent the possibility of systematic bias due to longitudinal scanner signal drift.

### DCE‐MRI protocol

We performed DCE‐MRI on a 3 T MR unit (Skyra, Siemens, Erlangen, Germany) using a 20‐element phased‐array head coil. For the dynamic sequence we used a 3D gradient echo sequence with TR = 2.48 ms, TE = 0.99 ms, flip angle = 15, linear phase ordering, GRAPPA undersampling with parallel imaging factor = 2, acquired matrix = 192 × 144 × 30, voxel size = 1.3 × 1.3 × 5.0 mm^3^, field of view = 250 × 188 × 150 mm^3^, reconstructed into 30 slices of thickness = 5.0 mm. The dynamic sequence comprised 300 dynamic frames at a time resolution of 3.2 s, giving a total scan duration of 16 min. Intravenous contrast injection was given after the 10th time point using an automated injector (Medrad; Newbury, UK), with speed 3 ml s^−1^, followed by a 30 ml saline flush at the same rate. Contrast agent was Gadovist (Bayer; Newbury, UK) at a dose of 0.05 mmol kg^−1^. We used half the standard clinical dose to avoid truncation artefacts of the bolus peak and provide an adequate washout curve (Taheri *et al*. 2011). In an initial optimization exercise, we performed a series of DCE‐MRI studies without injection of contrast agent, to estimate drift on our scanner over time; a linear fit of the trend in signal intensity over 15 min gave a mean drift of 0.2% min^−1^. This is comparable to previous studies by our group (Cramer *et al*. 2014) and others (Heye *et al*. 2016).

### MRI sequences

Initial measurement of T1 relaxation time was performed prior to the DCE sequence using a 3D gradient echo sequence with identical coverage and matrix, with a series of flip angles (5, 10, 15 and 18°), allowing calculation of baseline T1 using the variable flip angle approach, a well‐studied technique (Bojorquez *et al*. 2017). We used the same sequences for T1 mapping as for the dynamic acquisition, to avoid differences in B1. Prior to the dynamic acquisition we also performed a 3D magnetization‐prepared gradient echo (MP‐RAGE) sequence (TR = 2200 ms, TE = 2.45 ms, TI = 900 ms, flip angle = 8°, field‐of‐view 263 × 350 × 350 mm^3^, voxel size 1.0 × 1.0 × 1.0 mm^3^), axial T2‐weighted sequence (turbo spin echo; TR = 3600 ms, TE = 9.4 ms, field‐of‐view 263 × 350 × 350 mm^3^, voxel size 0.3 × 0.3 × 4.0 mm^3^, 35 slices), and coronal fluid‐attenuated inversion recovery (FLAIR) sequence (TR = 9000 ms, TE = 81 ms, TI = 2500 ms, field‐of‐view = 186 × 220 × 160 mm^3^, 40 slices). MP‐RAGE provides excellent contrast between grey and white matter and is widely used for tissue classification (Ashburner & Friston, 2000). After the dynamic acquisition, we performed a post‐contrast MP‐RAGE with identical parameters to the pre‐contrast image.

### Manual regions of interest

**Figure 1:**
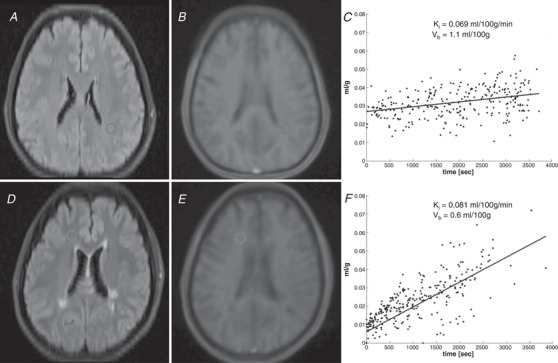
Region of interest (ROI) placement and Patlak plots *A*, axial fluid‐attenuated inversion recovery sequence (FLAIR) in a control subject with manual ROI placement in the normal‐appearing white matter. *B*, first dynamic frame from the same subject, with ROI transposed. *C*, Patlak plot derived from the ROI. *D–F*, same images for a subject with relapsing–remitting multiple sclerosis. ‘Time’ in the *x*‐axis of the Patlak plots is normalized to arterial concentration.

MS lesions were defined by T2 hyperintensity and typical location on FLAIR (Filippi *et al*. 2016). Non‐specific T2 hyperintense lesions in white matter were also excluded. Visibly contrast‐enhancing lesions (CELs) were identified through visual inspection of the post‐contrast T1 image. NAWM ROIs were drawn in the centrum semiovale on the axial FLAIR image (to distance from T2 hyperintense lesions), co‐registered to the post‐contrast T1 (to distance from CELs and grey matter), and then applied to the dynamic sequence. Co‐registration ensured that ROIs were placed >10 mm from MS lesions and >30 mm from CELs, both within slice and by reference to adjacent slices (see Fig. 1). Four NAWM ROIs were placed in total, two in each hemisphere, one anterior and one posterior to the central sulcus. In controls, grey matter ROIs were drawn in the thalami, one in each hemisphere. ROI size was standardized by visual inspection and intentionally aimed to capture the same volume of tissue as in our previous study (Cramer *et al*. 2014). The voxel count in ROIs was recorded to enable systematic examination for bias. ROI placement was performed by a single operator (A.V.). The operator could not be blinded to group due to the obvious presence of lesions in the RRMS cases, but the *K*_i_ values were only calculated after finalization of ROI placement, to minimize the potential for bias.

### Lesion detection

Lesions were segmented by the lesion growth algorithm (Schmidt *et al*. 2012) from LST version 20.0.15 (www.statistical-modelling.de/lst.html), operating within SPM version 12 (Ashburner & Friston, 2006). This algorithm first segments the isotropic T1 image into tissue classes, and then combines the information with co‐registered FLAIR intensities to calculate lesion belief maps. These maps were thresholded with a cut‐off (0.3) determined by visual inspection, to produce an initial binary lesion map. This was then grown along voxels that appear hyperintense in the FLAIR image, to produce a lesion probability map.

### Tissue segmentation

The high‐resolution T1 image was first brain‐extracted using BET (Smith, 2002). Tissue‐type segmentation was then performed using FAST (Zhang *et al*. 2001) and the resulting tissue probability map was transformed into the space of the dynamic images, so that thresholding of tissue probability could be performed in the DCE space. For RRMS cases, lesion filling was performed prior to segmentation using the LST toolbox. To ensure zero tolerance for partial volume artefact, a threshold of 100% was applied to the probability map to define the tissue mask. NAWM voxels were defined as those within the white matter mask with a zero value in the lesion probability map. All masks were quality‐controlled by visual inspection. Further analysis was performed on those slices covering the supratentorial brain (telencephalon).

### Tracer kinetic analysis

**Figure 2:**
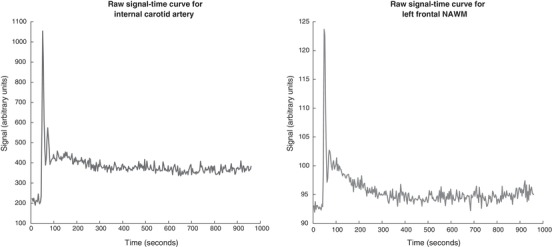
Representative signal‐time curves *A*, maximal signal change in the internal carotid arterial input function. *B*, mean behaviour of voxels from a tissue ROI in normal‐appearing white matter (NAWM). Both curves are from the same subject in Fig. 1 *D–F*.

We used custom‐built code in MATLAB (The Mathworks; Natick, MA, USA) to extract a signal–time series for each voxel (Fig. 2). The mean behaviour of voxels within either ROI (for the manual ROI method) or tissue mask (for the segmentation method) was used for further analysis; voxelwise analysis was computationally intensive and sensitive to noise. Baseline T1 combined with the contrast agent relaxivity of 4 s^−1^ mm ^−1^ (value quoted by the manufacturer) was used to convert the signal–time series into a concentration–time series. The arterial input function (AIF) was measured for each subject by determining the maximal signal change within an axial ROI drawn over the supraclinoid segment of the right internal carotid artery. The same AIF was used for both ROI and segmentation methods. *K*_i_ and CBV were calculated from the AIF and tissue concentration curve using the Patlak one‐tissue‐compartment model (Patlak *et al*. 1983). The necessary prerequisite for this model to be true is that the tracer is trapped ‘irreversibly’, meaning that significant back‐diffusion from tissue to blood does not occur during the measuring period. The Patlak equation is as follows:

$$\frac{C_{t}\left(t\right)}{C_{a}\left(t\right)}=K_{i}\frac{\int_{0}^{t}C_{a}\left(\tau \right)d\tau }{C_{a}\left(t\right)}+V_{b}$$

It plots the instantaneous tissue/arterial concentration ratio on the *y*‐axis and the integrated arterial concentration curve normalized to instantaneous arterial concentration on the *x*‐axis. The slope is *K*_i_, the volume of blood cleared of contrast per unit time. In a one‐tissue‐compartment model with no back diffusion, the intercept *V*_b_ is the CBV, as previously discussed (Larsson *et al*. 2009), so CBV is used in place of *V*_b_ throughout the paper. The linear part of the Patlak plot, i.e. the last two‐thirds of the data points, were included in the fitting procedure, to allow for steady state arterial concentration. Perfusion estimation was done by model‐free deconvolution of the tissue concentration with the arterial input function (using all data points), *ad modum* Tikhonov, which is a general form of singular value decomposition having a regularization term (Larsson *et al*. 2008). Values of *K*_i_ were reported as ml (100 g)^−1^ min^−1^, assuming brain tissue density of 1 g ml^−1^. In the ROI method, the mean of ROIs was quoted for each subject.

### Statistical analysis

Analysis was conducted in SPSS version 24 (IBM Corp., Armonk, NY, USA). Frequency distribution, normal probability plots and Kolmogorov–Smirnov testing were used to test normal distribution of raw or logarithmically transformed data. Appropriate two‐tailed tests were used to detect significance between groups for parametric or non‐parametric data. ANCOVA was used to compare RRMS and control *K*_i_, to enable inclusion of age as a scalar covariate. Multivariate linear regression was employed to examine CBV, CBF and tissue type as predictors of ROI‐determined *K*_i_. A *P*‐value of <0.05 allowed rejection of the null hypothesis. Bland–Altman analysis was used to assess agreement between the ROI and segmentation methods, as this has been shown to be the most appropriate tool for this purpose (Zaki *et al*. 2012). The intraclass correlation coefficient was used to assess reliability, incorporating both agreement and correlation (Koo & Li, 2016). All statistical analysis results are included in the Supporting information.

## Results

### Subjects

Thirteen control individuals and 12 patients with RRMS were recruited. Characteristics of participants are shown in Table 1. The RRMS group was older (*P* = 0.01, Student's *t* test), and age was therefore factored into all further analyses comparing groups, since BBB permeability increases with age (Elwood *et al*. 2017). The gender ratio between groups was not significantly different (*P* = 0.673, Fisher's exact test). The RRMS cohort was diverse, including both patients with early and patients with established disease, and those on and off treatment (Table 1). All RRMS subjects had typical MS lesions, and three patients had a total of four visibly contrast‐enhancing lesions (CELs). Tolerability of the scanning protocol was good, except for one RRMS subject who experienced discomfort towards the end of the session and required omission of the post‐contrast MP‐RAGE; for this subject all pre‐contrast sequences and the full DCE sequence were acquired and included in the analysis, but the presence of CELs could not be assessed.

**Table 1:** Characteristics of subjects

	HC (*n* = 13)	RRMS (*n* = 12)	*P*
Age (years)	31.08 (10.38)	42.75 (10.47)	0.01
Sex (% female)	61.5	75.0	0.673^a^
Disease duration (years)	–	10.75 (8.86)	
EDSS	–	2.29 (1.92)	
ROI size (voxels)	144 (16)	145 (23)	0.862
T2 lesion count	–	12.80 (7.45)	
T2 lesion volume (ml)	–	3.80 (3.47)	
Cases with CELs	–	3^b^	
Treatment type			
No treatment	13	4	
Interferon	–	4	
Glatiramer	–	1	
Fingolimod	–	2	
Alemtuzumab	–	1	

Values are mean (standard deviation). Difference in means is by unpaired *t* test, except in ^a^Fisher's exact test. ^b^One RRMS subject did not have a post‐contrast sequence for detection of CELs. CEL, contrast‐enhancing lesion; EDSS, Expanded Disability Status Score.

### Comparison of white and grey matter

**Figure 3:**
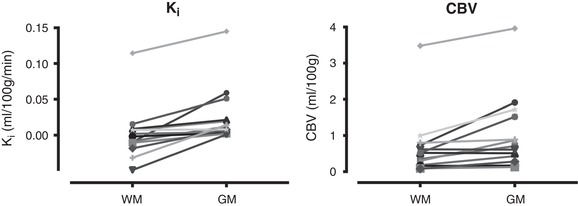
**Pairwise plots of *K*_i_ and CBV values in white matter (WM) and grey matter (GM), for individual control subjects**

*K*_i_ was significantly higher in grey matter than in white matter (*P* = 0.001, Wilcoxon) in healthy controls (Fig. 3). CBV was significantly higher in grey than white matter (*P* = 0.005, Wilcoxon), with a mean pair‐wise grey/white matter CBV ratio of 1.9 (range 1.0–3.6). A multivariate linear regression including ROI‐derived CBV, CBF and tissue type was performed to ascertain the extent to which these could explain *K*_i_ (full results in Supporting information, section 7). All variables were included in the final model, which explained 82% of the variance in *K*_i_ (*r* ^2^ = 0.816). Only CBV predicted *K*_i_ (β = 0.036, *P* < 10^−8^). Tissue type did not significantly predict *K*_i_ though a trend was observed (β = 0.015, *P* = 0.066). CBF did not correlate with *K*_i_ (Spearman's ρ = 0.32, *P* = 0.111).

### Multiple sclerosis

**Figure 4:**
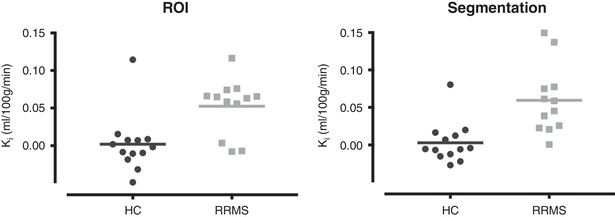
Scatterplot of *K*_i_ values in NAWM by either ROI or segmentation method Horizontal line is group mean.

Mean *K*_i_ in CELs (ROI method) was 0.139 ml (100 g)^−1^ min^−1^, significantly higher than NAWM in either RRMS (0.052 ml (100 g)^−1^ min^−1^, *P* = 0.002, *t* test) or controls (0.020 ml (100 g)^−1^ min^−1^, *P* = 0.005, Mann–Whitney). *K*_i_ in NAWM was significantly higher in RRMS than controls by both the ROI method (*P* = 0.014, ANCOVA, no effect of age) and the segmentation method (*P* = 0.019, ANCOVA, no effect of age). The results are shown in Table 2 and Fig. 4.

**Table 2:** Results for BBB permeability calculations in NAWM

	Measured values					Estimated marginal mean	
*K*_i_ (ml (100 g)^−1^ min^−1^)	HC (*n* = 13)	RRMS (*n* = 12)	*P*‐value for effect of group	Partial η^2^ for effect of group	*P*‐value for effect of age	HC (*n* = 13)	RRMS (*n* = 12)
ROI	0.020 (0.038)	0.052 (0.037)	0.014	0.246	0.789	0.003	0.051
Segmentation	0.003 (0.027)	0.045 (0.061)	0.019	0.226	0.090	0.010	0.052

Values are mean (standard deviation). Analysis is by ANCOVA incorporating age as a covariate.

### Comparison of ROI and segmentation methods

**Figure 5:**
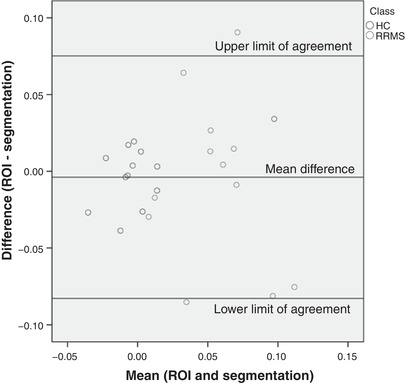
**Bland–Altman plot comparing ROI and segmentation methods**

A Bland–Altman plot comparing ROI and segmentation methods showed no clear difference between the calculated *K*_i_ values for NAWM. Full parameters are reported in Table 3. The difference in values by each method was not significantly different from zero (*P* = 0.638, one‐sample *t* test). Examination of the plot revealed no evidence of proportional bias according to *K*_i_ value (see Fig. 5). Accordingly, linear regression (with difference in *K*_i_ between the two methods as dependent, and mean *K*_i_ as predictor variable) confirmed absence of proportional bias (*P* = 0.859). The two‐way mixed intra‐class correlation coefficient (see Table 3) showed good single measure consistency (0.610).

**Table 3:** Comparison of ROI and segmentation methods and results of intra‐class correlation coefficient (ICC) with 95% confidence intervals

Parameter	Value
*n*	25
Minimum difference	−0.08523
Maximum difference	0.09050
Mean difference	−0.00384
Standard deviation of difference	0.04033
Upper 95% limit of agreement (95% confidence intervals)	0.07520 (0.04637 to 0.10404)
Lower 95% limit of agreement (95% confidence intervals)	−0.08288 (−0.11172 to −0.05405)
Difference between 95% limits of agreement	0.15809
ICC	0.610 (0.291–0.807)

## Discussion

This study supports the concept that DCE‐MRI can measure low‐level BBB leakage, since the influx constant *K*_i_ behaves according to the biological expectations of a marker of BBB leakage. It is higher in control GM compared to control WM, higher in MS NAWM compared to control WM and higher in CELs compared to NAWM. DCE‐MRI is capable of detecting BBB leakage more than an order of magnitude below the level found in CELs by conventional imaging.

Histological study in the human brain has shown that vascular volume is higher in GM than WM, with a mean GM/WM ratio of 2.9 (range 1.1–8.0) (Lierse & Horstmann, 1965). Other histological (Klein *et al*. 1986) as well as imaging studies (Leenders *et al*. 1990; Carroll *et al*. 2008) support this finding. If one assumes that the mean vessel radius is the same in GM and WM (in the absence of any evidence to the contrary), the GM/WM CBV ratio should be equal to the GM/WM vascular surface area ratio. If the influx constant *K*_i_ truly corresponds to the permeability‐surface area (*PS*) product, the larger vascular surface area in GM should contribute to a higher *K*_i_ in GM *versus* WM in the healthy brain. We show that the behaviour of *K*_i_ corresponds with that expected for the *PS* product in this regard. Other DCE‐MRI studies report conflicting findings: (1) higher GM CBV and *K*_i_ (Heye *et al*. 2016), (2) higher GM *K*_i_ (Cramer *et al*. 2014), (3) higher GM CBV but lower *K*_i_ (van de Haar *et al*. 2016), and (4) lower GM *K*_i_ (Montagne *et al*. 2015).

In healthy controls, we find that CBV, which scales with surface area, predicts *K*_i_. When controlling for CBV, CBF and tissue type did not significantly predict *K*_i_. A trend for tissue type (*P* = 0.066) was seen, but only explained 2% of the variance in *K*_i_, when CBV explained 63% of the variance in *K*_i_ in an ANCOVA (Supporting information, section 8). This could be due to minor differences in permeability or vessel radius between GM and WM, and should be investigated further.

According to the Crone–Renkin equation (Renkin, 1959; Crone, 1963), when CBF is much larger than the *PS* product (which is the case at the BBB), *K*_i_ approximates the *PS* product and there should not be a correlation between CBF and *K*_i_. In keeping with this theory, we do not observe a correlation between CBF and *K*_i_, which is additional supporting evidence for the validity of the method.

This study has some limitations. Firstly, we used a half‐dose of contrast agent, which reduces the contrast‐to‐noise ratio and impairs detection of enhancing lesions (Rovira *et al*. 2015). This means that our definition of NAWM could have been confounded by the presence of undetected enhancing lesions. However, the choice of half‐dose was based on a trade‐off between increasing contrast‐to‐noise ratio, and introducing truncation artefact to the bolus peak and/or T2^*^ effects at high concentrations. We have reported good results with half‐dose injection in numerous studies (Larsson *et al*. 2009; Cramer et al. 2014, 2015, 2018).

Secondly, there were potential sources of error in the acquisition. B1 inhomogeneity can lead to errors in baseline T1 estimation and therefore the conversion of dynamic signal to concentration. However, as we used the same sequence for T1 measurement and dynamic run, any such bias is constant between the two, so that B1 errors in T1 mapping and DCE will tend to mutually cancel. Also, we used M0 values derived from the T1 measurement when converting signal to concentration, which would also tend to compensate for B1 inhomogeneity. We measured a background signal drift that was comparable to previous studies (Cramer *et al*. 2014; Heye *et al*. 2016). The causes of signal drift are poorly understood. Signal drift affects the accuracy of absolute measurements of *K*_i_, but this is less of an issue in studies with control groups, where comparison is made between diseased and healthy subjects, or between experimental and non‐experimental situations. Future studies should examine methods for measuring and correcting for signal drift, ideally in a subject‐ and tissue‐specific fashion.

Thirdly, we measured a carotid AIF, which has advantages and disadvantages compared to a venous ‘input’ function (VIF) measured in, for example, the sagittal sinus. Theoretically the AIF represents a true input, unlike the VIF. The curve shape and peak sharpness of the AIF and VIF may be different. The time delay on the venous curve may not influence the Patlak calculation of *K*_i_ but will have a significant impact on the estimation of CBF. On the other hand the AIF is more prone to partial volume and the in‐flow phenomenon. In practice, choice of AIF or VIF does not seem to significantly impact the *K*_i_, as shown by one published study (Filice & Crisi, 2016) and work of our own (unpublished, in preparation). In the present study, we have minimized partial volume effects by selecting the voxel with maximum signal change during bolus passage in the AIF ROI, and we have used a relatively high acquired spatial resolution.

Fourthly, the FLAIR sequences used were 2D, which is inferior to 3D in the detection of MS lesions (Bink *et al*. 2006). The automated lesion detection algorithm is validated for 3D FLAIR (Egger *et al*. 2017). Thus we could have inadvertently included lesional tissue in NAWM during segmentation. However, we avoided this by visually inspecting all the masks. Also, this would have affected the ROI method to a much lesser extent, but we observed similar results.

Fifthly, age was significantly higher in RRMS *versus* control groups. Increasing age is associated with higher BBB permeability (Montagne *et al*. 2015; Elwood *et al*. 2017; Erdö *et al*. 2017; Bors *et al*. 2018), so this was a possible confounder. However, age was included as a covariate, and a significant effect of age on *K*_i_ was not observed in this small dataset (see Table 2).

Inter‐species differences in BBB structure and function limit the applicability of animal studies to humans (Deo *et al*. 2013), and *in vitro* models cannot fully recapitulate the natural complexity of the BBB (Naik & Cucullo, 2012). Human studies have been hampered by the lack of a validated non‐invasive method for the quantification of BBB permeability. This study provides incremental evidence to increase confidence in the interpretation of *K*_i_ derived from DCE‐MRI as a BBB permeability marker in research and clinical applications. Comparing *K*_i_ with CSF/serum albumin ratio (*Q*_alb_) may be another way to cross‐validate these two markers of BBB leakage. In one study, hippocampal *K*_i_ correlated with *Q*_alb_ (Montagne *et al*. 2015) but WM *K*_i_ did not correlate with CSF/serum albumin ratio in another study (Taheri *et al*. 2011). It is hard to draw conclusions since *Q*_alb_ integrates BBB leakage across the whole neural axis, and the relative timing of lumbar puncture and DCE‐MRI was not stated in these studies. It is possible that DCE‐MRI is measuring gadolinium adhering to the luminal surface of cerebral capillary endothelial cells at the BBB, as opposed to transendothelial permeation, but this is unlikely since gadolinium can be detected in the neuronal interstitium itself (McDonald *et al*. 2015). Future studies may use DCE‐MRI to explore (1) physiological variation in BBB function related to variables such as age (Elwood *et al*. 2017) or sex (Maggioli *et al*. 2015); (2) BBB changes in pathology; (3) the effect of different physiological states on the BBB; and (4) the evaluation of pharmacological agents which prevent or reverse BBB disruption.

It has already been shown that measurement of BBB leakage with DCE‐MRI has moderate‐to‐excellent reproducibility (Wong *et al*. 2017). However there are hurdles restricting the widespread use of DCE‐MRI. Factors such as scanner field strength, careful detection of baseline pre‐contrast signal (Barnes *et al*. 2016), use of individual AIFs for each subject, partial volume correction of the AIF (Hansen *et al*. 2009), selecting the optimal time resolution and total scan time (Cramer & Larsson, 2014), and choosing a suitable pharmacokinetic modelling approach (Sourbron & Buckley, 2013) appear to be important for accurate estimation of subtle BBB leakage. Another hurdle is the time and skill required for manual ROI placement and the potential for operator bias. We show how automated tissue segmentation eliminates the need for manual ROI placement, capturing more diffuse abnormality whilst reducing operator dependence and providing ease of batch processing. If we take the mean difference between controls and RRMS to be meaningful, then the difference between 95% limits of agreement exceeded this level, so the ROI and segmentation methods cannot be used interchangeably. However, the two methods show good agreement in ratio of change, as evidenced by the ICC. Another hurdle is reproducibility of results between scanners. However, multi‐centre studies using DCE‐MRI might be feasible if the study outcome is the ratio of change in *K*_i_ (in the case of longitudinal *K*_i_ measurements, e.g. before and after treatment) or inter‐group *K*_i_ ratios (in the case of controlled studies, e.g. placebo and active drug). Such an approach would be particularly suitable for studies assessing the effect of therapeutics targeted at the BBB.

### Conclusion

We provide evidence for the validity of *K*_i_ derived from three‐dimensional DCE‐MRI as a marker of overall BBB leakage, assessed against the following criteria: (1) higher *K*_i_ in control grey *versus* white matter, (2) grey/white matter vascular surface area ratio close to the histologically established value, (3) higher *K*_i_ in contrast‐enhancing white matter lesions *versus* RRMS NAWM, and (4) higher *K*_i_ in MS NAWM *versus* control WM. By incorporating 3D imaging, we enable automated tissue segmentation, and we demonstrate that the DCE‐MRI protocol is robust to segmentation. This will help reduce operator bias and facilitate uptake of DCE‐MRI for further study of the human BBB.

## Additional information

Detailed results of all the statistical analyses.

### Competing interests

A.V. has received funding for travel and conference attendance from Teva and Ipsen. S.C. has received research funding and honoraria for speaking engagements and travel from Biogen Idec, honoraria for speaking engagements from Genzyme, and funding for travel from Merck Serono and Roche. H.L. has received research funding from Biogen Idec. I.G. has received funding for travel and conference attendance from Teva, research funding from Merck Serono and Evgen, and has served as a scientific advisor to Evgen. M.L. and A.D. have no conflicts of interest to report.

### Author contributions

Design of the work: A.V., I.G.; acquisition, analysis and interpretation of data: A.V., M.L., A.D., H.L., I.G. and S.C.; drafting the work: A.V.; critical revision for important intellectual content: H.L., I.G. and S.C. All authors approved the final version of manuscript and agree to be accountable for all aspects of the work in ensuring that questions related to the accuracy or integrity of any part of the work are appropriately investigated and resolved. All persons designated as authors qualify for authorship, and all those who qualify for authorship are listed.

### Funding

This project was supported by the University of Southampton, National Institute of Health Research (award reference 3117), Medical Research Council (award reference MR/R017352/1), and MS Society (award reference 996).

## Supporting information

Detailed results of all the statistical analyses.

## References

[tjp13333-bib-0001] Abbott NJ , Patabendige AAK , Dolman DEM , Yusof SR & Begley DJ (2010). Structure and function of the blood‐brain barrier. Neurobiol Dis 37, 13–25. 19664713 10.1016/j.nbd.2009.07.030

[tjp13333-bib-0002] Ashburner J & Friston KJ (2000). Voxel‐based morphometry—the methods. Neuroimage 11, 805–821. 10860804 10.1006/nimg.2000.0582

[tjp13333-bib-0020] Ashburner JT & Friston KJ (2006). Segmentation In Statistical Parametric Mapping: The Analysis of Functional Brain Images, eds. Friston KJ, Ashburner JT, Kieber SJ, Nichols TE & Penny WD, pp. 81–91.

[tjp13333-bib-0003] Barnes SR , Ng TSC , Montagne A , Law M , Zlokovic B V . & Jacobs RE (2016). Optimal acquisition and modeling parameters for accurate assessment of low K_trans_ blood‐brain barrier permeability using dynamic contrast‐enhanced MRI. Magn Reson Med 75, 1967–1977. 26077645 10.1002/mrm.25793 PMC4726482

[tjp13333-bib-0004] Bechmann I , Galea I & Perry VH (2007). What is the blood‐brain barrier (not)? Trends Immunol 28, 5–11. 17140851 10.1016/j.it.2006.11.007

[tjp13333-bib-0005] Bink A , Schmitt M , Gaa J , Mugler JP , Lanfermann H & Zanella FE (2006). Detection of lesions in multiple sclerosis by 2 D FLAIR and single‐slab 3 D FLAIR sequences at 3.0T: Initial results. Eur Radiol 16, 1104–1110. 16425026 10.1007/s00330-005-0107-z

[tjp13333-bib-0006] Bojorquez JZ , Bricq S , Acquitter C , Brunotte F , Walker PM & Lalande A (2017). What are normal relaxation times of tissues at 3T? Magn Reson Imaging 35, 69–80. 27594531 10.1016/j.mri.2016.08.021

[tjp13333-bib-0007] Bors L , Tóth K , Tóth EZ , Bajza Á , Csorba A , Szigeti K , Máthé D , Perlaki G , Orsi G , Tóth GK & Erdő F (2018). Age‐dependent changes at the blood‐brain barrier. A Comparative structural and functional study in young adult and middle aged rats. Brain Res Bull 139, 269–277. 29522862 10.1016/j.brainresbull.2018.03.001

[tjp13333-bib-0008] Carroll TJ , Horowitz S , Shin W , Mouannes J , Sawlani R , Ali S , Raizer J & Futterer S (2008). Quantification of cerebral perfusion using the “bookend technique”: an evaluation in CNS tumors. Magn Reson Imaging 26, 1352–1359. 18538523 10.1016/j.mri.2008.04.010 PMC2612563

[tjp13333-bib-0009] Cramer SP & Larsson HBW (2014). Accurate determination of blood‐brain barrier permeability using dynamic contrast‐enhanced T1‐weighted MRI: a simulation and in vivo study on healthy subjects and multiple sclerosis patients. J Cereb Blood Flow Metab 34, 1655–1665. 25074746 10.1038/jcbfm.2014.126 PMC4269724

[tjp13333-bib-0010] Cramer SP , Modvig S , Simonsen HJ , Frederiksen JL & Larsson HBW (2015). Permeability of the blood‐brain barrier predicts conversion from optic neuritis to multiple sclerosis. Brain 138, 2571–2583. 26187333 10.1093/brain/awv203 PMC4547053

[tjp13333-bib-0011] Cramer SP , Simonsen H , Frederiksen JL , Rostrup E & Larsson HBW (2014). Abnormal blood‐brain barrier permeability in normal appearing white matter in multiple sclerosis investigated by MRI. Neuroimage Clin 4, 182–189. 24371801 10.1016/j.nicl.2013.12.001 PMC3872721

[tjp13333-bib-0012] Cramer SP , Simonsen HJ , Varatharaj A , Galea I , Frederiksen JL & Larsson HBW (2018). Permeability of the blood‐brain barrier predicts no evidence of disease activity at two years after natalizumab or fingolimod treatment in relapsing‐remitting multiple sclerosis. Ann Neurol 83, 902–914. 29604233 10.1002/ana.25219 PMC6032831

[tjp13333-bib-0013] Crone C (1963). The permeability of capillaries in various organs as determined by use of the ‘indicator diffusion’ method. Acta Physiol Scand 58, 292–305. 14078649 10.1111/j.1748-1716.1963.tb02652.x

[tjp13333-bib-0014] Deo AK , Theil FP & Nicolas JM (2013). Confounding parameters in preclinical assessment of blood‐brain barrier permeation: An overview with emphasis on species differences and effect of disease states. Mol Pharm 10, 1581–1595. 23256608 10.1021/mp300570z

[tjp13333-bib-0015] Egger C , Opfer R , Wang C , Kepp T , Sormani MP , Spies L , Barnett M & Schippling S (2017). MRI FLAIR lesion segmentation in multiple sclerosis: Does automated segmentation hold up with manual annotation? Neuroimage Clin 13, 264–270. 28018853 10.1016/j.nicl.2016.11.020 PMC5175993

[tjp13333-bib-0016] Elwood E , Lim Z , Naveed H & Galea I (2017). The effect of systemic inflammation on human brain barrier function. Brain Behav Immun 62, 35–40. 27810376 10.1016/j.bbi.2016.10.020 PMC5380128

[tjp13333-bib-0017] Erdö F , Denes L & De Lange E (2017). Age‐associated physiological and pathological changes at the blood‐brain barrier: A review. J Cereb Blood Flow Metab 37, 4–24. 27837191 10.1177/0271678X16679420 PMC5363756

[tjp13333-bib-0018] Filice S & Crisi G (2016). Dynamic contrast‐enhanced perfusion MRI of high grade brain gliomas obtained with arterial or venous waveform input function. J Neuroimaging 26, 124–129. 25923172 10.1111/jon.12254

[tjp13333-bib-0019] Filippi M , Rocca MA , Ciccarelli O , De Stefano N , Evangelou N , Kappos L , Rovira A , Sastre‐Garriga J , Tintor M , Frederiksen JL , Gasperini C , Palace J , Reich DS , Banwell B , Montalban X & Barkhof F (2016). MRI criteria for the diagnosis of multiple sclerosis: MAGNIMS consensus guidelines. Lancet Neurol 15, 292–303. 26822746 10.1016/S1474-4422(15)00393-2 PMC4760851

[tjp13333-bib-0021] Hansen AE , Pedersen H , Rostrup E & Larsson HBW (2009). Partial volume effect (PVE) on the arterial input function (AIF) in T 1‐weighted perfusion imaging and limitations of the multiplicative rescaling approach. Magn Reson Med 62, 1055–1059. 19672948 10.1002/mrm.22098

[tjp13333-bib-0022] Heye AK , Thrippleton MJ , Armitage PA , Valdés Hernández MDC , Makin SD , Glatz A , Sakka E & Wardlaw JM (2016). Tracer kinetic modelling for DCE‐MRI quantification of subtle blood‐brain barrier permeability. Neuroimage 125, 446–455. 26477653 10.1016/j.neuroimage.2015.10.018 PMC4692516

[tjp13333-bib-0023] Iannotti F , Fieschi C , Alfano B , Picozzi P , Mansi L , Pozzilli C , Punzo A , Del Vecchio G , Lenzi GL , Salvatore M & Conforti P (1987). Simplified, noninvasive PET measurement of blood‐brain barrier permeability. J Comput Assist Tomogr 11, 390–397. 3106433 10.1097/00004728-198705000-00004

[tjp13333-bib-0024] Iliff JJ , Wang M , Liao Y , Plogg BA , Peng W , Gundersen GA , Benveniste H , Vates GE , Deane R , Goldman SA , Nagelhus EA & Nedergaard M (2012). A paravascular pathway facilitates CSF flow through the brain parenchyma and the clearance of interstitial solutes, including amyloid. Sci Transl Med 4, 147ra111. 10.1126/scitranslmed.3003748 PMC3551275 22896675

[tjp13333-bib-0025] Jost G , Frenzel T , Lohrke J , Lenhard DC , Naganawa S & Pietsch H (2017). Penetration and distribution of gadolinium‐based contrast agents into the cerebrospinal fluid in healthy rats: a potential pathway of entry into the brain tissue. Eur Radiol 27, 2877–2885. 27832312 10.1007/s00330-016-4654-2 PMC5486780

[tjp13333-bib-0026] Kirk J , Plumb J , Mirakhur M & McQuaid S (2003). Tight junctional abnormality in multiple sclerosis white matter affects all calibres of vessel and is associated with blood‐brain barrier leakage and active demyelination. J Pathol 201, 319–327. 14517850 10.1002/path.1434

[tjp13333-bib-0027] Klein B , Kuschinsky W , Schröck H & Vetterlein F (1986). Interdependency of local capillary density, blood flow, and metabolism in rat brains. Am J Physiol 251, H1333–H1340. 3098116 10.1152/ajpheart.1986.251.6.H1333

[tjp13333-bib-0028] Koo TK & Li MY (2016). A guideline of selecting and reporting intraclass correlation coefficients for reliability research. J Chiropr Med 15, 155–163. 27330520 10.1016/j.jcm.2016.02.012 PMC4913118

[tjp13333-bib-0029] Larsson HBW , Courivaud F , Rostrup E & Hansen AE (2009). Measurement of brain perfusion, blood volume, and blood‐brain barrier permeability, using dynamic contrast‐enhanced T1‐weighted MRI at 3 tesla. Magn Reson Med 62, 1270–1281. 19780145 10.1002/mrm.22136

[tjp13333-bib-0030] Larsson HBW , Hansen AE , Berg HK , Rostrup E & Haraldseth O (2008). Dynamic contrast‐enhanced quantitative perfusion measurement of the brain using T1‐weighted MRI at 3T. J Magn Reson Imaging 27, 754–762. 18383268 10.1002/jmri.21328

[tjp13333-bib-0031] Leenders KL , Perani D , Lammertsma AA , Heather JD , Buckingham P , Jones T , Healy MJR , Gibbs JM , Wise RJS , Hatazawa J , Herold S , Beaney RP , Brooks DJ , Spinks T , Rhodes C & Frackowiak RSJ (1990). Cerebral blood flow, blood volume and oxygen utilization: Normal values and effect of age. Brain 113, 27–47. 2302536 10.1093/brain/113.1.27

[tjp13333-bib-0032] Lierse W & Horstmann E (1965). Quantitative anatomy of the cerebral vascular bed with especial emphasis on homogeneity and inhomogeneity in small parts of the gray and white matter. Acta Neurol Scand 41, 15–19. 10.1111/j.1600-0404.1965.tb01946.x 5214090

[tjp13333-bib-0033] Lund H , Krakauer M , Skimminge A , Sellebjerg F , Garde E , Siebner HR , Paulson OB , Hesse D & Hanson LG (2013). Blood‐brain barrier permeability of normal appearing white matter in relapsing‐remitting multiple sclerosis. PLoS One 8, e56375. 23441184 10.1371/journal.pone.0056375 PMC3575471

[tjp13333-bib-0034] Maggioli E , McArthur S , Mauro C , Kieswich J , Kusters DHM , Reutelingsperger CPM , Yaqoob M & Solito E (2015). Estrogen protects the blood‐brain barrier from inflammation‐induced disruption and increased lymphocyte trafficking. Brain Behav Immun 51, 212–222. 26321046 10.1016/j.bbi.2015.08.020

[tjp13333-bib-0035] McDonald RJ , McDonald JS , Kallmes DF , Jentoft ME , Murray DL , Thielen KR , Williamson EE & Eckel LJ (2015). Intracranial gadolinium deposition after contrast‐enhanced MR imaging. Radiology 275, 772–782. 25742194 10.1148/radiol.15150025

[tjp13333-bib-0036] Montagne A , Barnes SR , Sweeney MD , Halliday MR , Sagare AP , Zhao Z , Toga AW , Jacobs RE , Liu CY , Amezcua L , Harrington MG , Chui HC , Law M & Zlokovic BV (2015). Blood‐brain barrier breakdown in the aging human hippocampus. Neuron 85, 296–302. 25611508 10.1016/j.neuron.2014.12.032 PMC4350773

[tjp13333-bib-0037] Naik P & Cucullo L (2012). In vitro blood‐brain barrier models: Current and perspective technologies. J Pharm Sci 101, 1337–1354. 22213383 10.1002/jps.23022 PMC3288147

[tjp13333-bib-0038] Patlak CS , Blasberg RG & Fenstermacher JD (1983). Graphical evaluation of blood‐to‐brain transfer constants from multiple‐time uptake data. J Cereb Blood Flow Metab 3, 1–7. 6822610 10.1038/jcbfm.1983.1

[tjp13333-bib-0039] Reiber H (1994). Flow rate of cerebrospinal fluid (CSF) – A concept common to normal blood‐CSF barrier function and to dysfunction in neurological diseases. J Neurol Sci 122, 189–203. 8021703 10.1016/0022-510x(94)90298-4

[tjp13333-bib-0040] Renkin E (1959). Transport of potassium‐42 from blood to tissue in isolated mammalian skeletal muscles. Am J Physiol 197, 1205–1210. 14437359 10.1152/ajplegacy.1959.197.6.1205

[tjp13333-bib-0041] Rovira À , Wattjes MP , Tintoré M , Tur C , Yousry TA , Sormani MP , De Stefano N , Filippi M , Auger C , Rocca MA , Barkhof F , Fazekas F , Kappos L , Polman C , Miller D , Montalban X ; MAGNIMS study group (2015). Evidence‐based guidelines: MAGNIMS consensus guidelines on the use of MRI in multiple sclerosis – clinical implementation in the diagnostic process. Nat Rev Neurol 11, 471‐4882. 26149978 10.1038/nrneurol.2015.106

[tjp13333-bib-0042] Schmidt P , Gaser C , Arsic M , Buck D , Förschler A , Berthele A , Hoshi M , Ilg R , Schmid VJ , Zimmer C , Hemmer B & Mühlau M (2012). An automated tool for detection of FLAIR‐hyperintense white‐matter lesions in Multiple Sclerosis. Neuroimage 59, 3774–3783. 22119648 10.1016/j.neuroimage.2011.11.032

[tjp13333-bib-0043] Silver NC , Tofts PS , Symms MR , Barker GJ , Thompson AJ , Miller DH (2001). Quantitative contrast‐enhanced magnetic resonance imaging to evaluate blood‐brain barrier integrity in multiple sclerosis: A preliminary study. Mult Scler 7, 75–82. 11424635 10.1177/135245850100700201

[tjp13333-bib-0044] Smith SM (2002). Fast robust automated brain extraction. Hum Brain Mapp 17, 143–155. 12391568 10.1002/hbm.10062 PMC6871816

[tjp13333-bib-0045] Sourbron SP & Buckley DL (2013). Classic models for dynamic contrast‐enhanced MRI. NMR Biomed 26, 1004–1027. 23674304 10.1002/nbm.2940

[tjp13333-bib-0046] Taheri S , Gasparovic C , Shah NJ & Rosenberg GA (2011). Quantitative measurement of blood‐brain barrier permeability in human using dynamic contrast‐enhanced MRI with fast T1 mapping. Magn Reson Med 65, 1036–1042. 21413067 10.1002/mrm.22686 PMC4950947

[tjp13333-bib-0047] Taheri S , Rosenberg GA & Ford C (2013). Quantification of blood‐to‐brain transfer rate in multiple sclerosis. Mult Scler Relat Disord 2, 124–132. 25877634 10.1016/j.msard.2012.09.003 PMC4874521

[tjp13333-bib-0048] van de Haar HJ , Burgmans S , Jansen JFA , van Osch MJP , van Buchem MA , Muller M , Hofman PAM , Verhey FRJ & Backes WH (2016). Blood‐brain barrier leakage in patients with early Alzheimer disease. Radiology 282, 615–615. 10.1148/radiol.2017164043 28099097

[tjp13333-bib-0049] Vos CMP , Geurts JJG , Montagne L , Van Haastert ES , Bö L , Van Der Valk P , Barkhof F & De Vries HE (2005). Blood‐brain barrier alterations in both focal and diffuse abnormalities on postmortem MRI in multiple sclerosis. Neurobiol Dis 20, 953–960. 16039866 10.1016/j.nbd.2005.06.012

[tjp13333-bib-0050] Wong SM , Jansen JFA , Zhang CE , Staals J , Hofman PAM , van Oostenbrugge RJ , Jeukens CRLPN & Backes WH (2017). Measuring subtle leakage of the blood‐brain barrier in cerebrovascular disease with DCE‐MRI: Test‐retest reproducibility and its influencing factors. J Magn Reson Imaging 46, 159–166. 28160347 10.1002/jmri.25540

[tjp13333-bib-0051] Zaki R , Bulgiba A , Ismail R & Ismail NA (2012). Statistical methods used to test for agreement of medical instruments measuring continuous variables in method comparison studies: A systematic review. PLoS One 7, e37908. 22662248 10.1371/journal.pone.0037908 PMC3360667

[tjp13333-bib-0052] Zhang Y , Brady M & Smith S (2001). Segmentation of brain MR images through a hidden Markov random field model and the expectation‐maximization algorithm. IEEE T Med Imaging 20, 45–57. 10.1109/42.906424 11293691

