# Supplementary material for: Blood–brain barrier permeability measured using dynamic contrast‐enhanced magnetic resonance imaging: a validation study
Source: J Physiol. 2018 Nov 29;597(3):699–709. doi: 10.1113/JP276887 (PMC6355631; doi:10.1113/JP276887)
Supplement: Supplementary file 1 — Detailed results of all the statistical analyses. [file TJP-597-699-s001.docx]

**Supporting information**

**1. Comparison of subject age between control and RRMS groups (t-test)**

| **Group Statistics** | | | | | |
| --- | --- | --- | --- | --- | --- |
|  | Class | N | Mean | Std. Deviation | Std. Error Mean |
| Age | HC | 13 | 31.08 | 10.380 | 2.879 |
|  | RRMS | 12 | 42.75 | 10.446 | 3.015 |

| **Independent Samples Test** | | | | | | | |
| --- | --- | --- | --- | --- | --- | --- | --- |
|  | | Levene's Test for Equality of Variances | | t-test for Equality of Means | | | |
|  |  | F | Sig. | t | df | Sig. (2-tailed) | Mean Difference |
|  |  |  |  |  |  |  |  |
| Age | Equal variances assumed | .392 | .537 | -2.801 | 23 | .010 | -11.673 |
|  | Equal variances not assumed |  |  | -2.800 | 22.816 | .010 | -11.673 |

| **Independent Samples Test** | | | | |
| --- | --- | --- | --- | --- |
|  | | t-test for Equality of Means | | |
|  |  | Std. Error Difference | 95% Confidence Interval of the Difference | |
|  |  |  | Lower | Upper |
| Age | Equal variances assumed | 4.168 | -20.295 | -3.051 |
|  | Equal variances not assumed | 4.169 | -20.301 | -3.045 |

**2. Comparison of subject gender between control and RRMS groups (crosstabs)**

| **Case Processing Summary** | | | | | | |
| --- | --- | --- | --- | --- | --- | --- |
|  | Cases | | | | | |
|  | Valid | | Missing | | Total | |
|  | N | Percent | N | Percent | N | Percent |
| Class * Gender | 25 | 86.2% | 4 | 13.8% | 29 | 100.0% |

| **Class * Gender Crosstabulation** | | | | | |
| --- | --- | --- | --- | --- | --- |
|  | | | Gender | | Total |
|  |  |  | Male | Female |  |
| Class | HC | Count | 5 | 8 | 13 |
|  |  | % within Class | 38.5% | 61.5% | 100.0% |
|  |  | % within Gender | 62.5% | 47.1% | 52.0% |
|  | RRMS | Count | 3 | 9 | 12 |
|  |  | % within Class | 25.0% | 75.0% | 100.0% |
|  |  | % within Gender | 37.5% | 52.9% | 48.0% |
| Total | | Count | 8 | 17 | 25 |
|  |  | % within Class | 32.0% | 68.0% | 100.0% |
|  |  | % within Gender | 100.0% | 100.0% | 100.0% |

| **Chi-Square Tests** | | | | | |
| --- | --- | --- | --- | --- | --- |
|  | Value | df | Asymptotic Significance (2-sided) | Exact Sig. (2-sided) | Exact Sig. (1-sided) |
| Pearson Chi-Square | .520^a^ | 1 | .471 |  |  |
| Continuity Correction^b^ | .085 | 1 | .770 |  |  |
| Likelihood Ratio | .524 | 1 | .469 |  |  |
| Fisher's Exact Test |  |  |  | .673 | .387 |
| Linear-by-Linear Association | .499 | 1 | .480 |  |  |
| N of Valid Cases | 25 |  |  |  |  |

**3. Comparison of ROI size between control and RRMS groups (T-Test)**

| **Group Statistics** | | | | | |
| --- | --- | --- | --- | --- | --- |
|  | Class | N | Mean | Std. Deviation | Std. Error Mean |
| ROI_size | HC | 13 | 144.3846 | 16.00280 | 4.43838 |
|  | RRMS | 12 | 145.7500 | 22.51111 | 6.49840 |

| **Independent Samples Test** | | | | | | |
| --- | --- | --- | --- | --- | --- | --- |
|  | | Levene's Test for Equality of Variances | | t-test for Equality of Means | | |
|  |  | F | Sig. | t | df | Sig. (2-tailed) |
|  |  |  |  |  |  |  |
| ROI_size | Equal variances assumed | .890 | .355 | -.176 | 23 | .862 |
|  | Equal variances not assumed |  |  | -.174 | 19.722 | .864 |

| **Independent Samples Test** | | | | | |
| --- | --- | --- | --- | --- | --- |
|  | | t-test for Equality of Means | | | |
|  |  | Mean Difference | Std. Error Difference | 95% Confidence Interval of the Difference | |
|  |  |  |  | Lower | Upper |
| ROI_size | Equal variances assumed | -1.36538 | 7.76220 | -17.42271 | 14.69195 |
|  | Equal variances not assumed | -1.36538 | 7.86946 | -17.79562 | 15.06485 |

**4. Comparison of paired grey and white matter K_i_ (Wilcoxon)**

**Wilcoxon Signed Ranks Test**

| **Ranks** | | | | |
| --- | --- | --- | --- | --- |
|  | | N | Mean Rank | Sum of Ranks |
| GM_Ki_ROI - WM_Ki_ROI | Negative Ranks | 0^a^ | .00 | .00 |
|  | Positive Ranks | 13^b^ | 7.00 | 91.00 |
|  | Ties | 0^c^ |  |  |
|  | Total | 13 |  |  |

| a. GM_Ki_ROI < WM_Ki_ROI |
| --- |
| b. GM_Ki_ROI > WM_Ki_ROI |
| c. GM_Ki_ROI = WM_Ki_ROI |

| **Test Statistics^a^** | |
| --- | --- |
|  | GM_Ki_ROI - WM_Ki_ROI |
| Z | -3.180^b^ |
| Asymp. Sig. (2-tailed) | .001 |

| a. Wilcoxon Signed Ranks Test |
| --- |
| b. Based on negative ranks. |

**5. Comparison of paired grey and white matter CBV (Wilcoxon)**

**Wilcoxon Signed Ranks Test**

| **Ranks** | | | | |
| --- | --- | --- | --- | --- |
|  | | N | Mean Rank | Sum of Ranks |
| GM_CBVol_ROI - WM_CBVol_ROI | Negative Ranks | 2^a^ | 2.50 | 5.00 |
|  | Positive Ranks | 11^b^ | 7.82 | 86.00 |
|  | Ties | 0^c^ |  |  |
|  | Total | 13 |  |  |

| a. GM_CBVol_ROI < WM_CBVol_ROI |
| --- |
| b. GM_CBVol_ROI > WM_CBVol_ROI |
| c. GM_CBVol_ROI = WM_CBVol_ROI |

| **Test Statistics^a^** | |
| --- | --- |
|  | GM_CBVol_ROI - WM_CBVol_ROI |
| Z | -2.830^b^ |
| Asymp. Sig. (2-tailed) | .005 |

| a. Wilcoxon Signed Ranks Test |
| --- |
| b. Based on negative ranks. |

**6. Pair-wise grey/white matter CBV ratio**

| **Descriptive Statistics** | | | | | |
| --- | --- | --- | --- | --- | --- |
|  | N | Minimum | Maximum | Mean | Std. Deviation |
| GM‎_WM_ratio | 13 | .97 | 3.64 | 1.9117 | .90536 |
| Valid N (listwise) | 13 |  |  |  |  |

7. **Regression of factors influencing K_i_**

| **Variables Entered/Removed^a^** | | | |
| --- | --- | --- | --- |
| Model | Variables Entered | Variables Removed | Method |
| 1 | CBF, CBV, Tissue^b^ | . | Enter |

| a. Dependent Variable: Ki |
| --- |
| b. All requested variables entered. |

| **Model Summary** | | | | |
| --- | --- | --- | --- | --- |
| Model | R | R Square | Adjusted R Square | Std. Error of the Estimate |
| 1 | .903^a^ | .816 | .791 | .018436525 |

| a. Predictors: (Constant), CBF, CBV, Tissue |
| --- |

| **ANOVA^a^** | | | | | | |
| --- | --- | --- | --- | --- | --- | --- |
| Model | | Sum of Squares | df | Mean Square | F | Sig. |
| 1 | Regression | .033 | 3 | .011 | 32.450 | .000^b^ |
|  | Residual | .007 | 22 | .000 |  |  |
|  | Total | .041 | 25 |  |  |  |

| a. Dependent Variable: Ki |
| --- |
| b. Predictors: (Constant), CBF, CBV, Tissue |

| **Coefficients^a^** | | | | | | | | |
| --- | --- | --- | --- | --- | --- | --- | --- | --- |
| Model | | Unstandardized Coefficients | | Standardized Coefficients | t | Sig. | 95.0% Confidence Interval for B | |
|  |  | B | Std. Error | Beta |  |  | Lower Bound | Upper Bound |
| 1 | (Constant) | -.018 | .006 |  | -2.739 | .012 | -.031 | -.004 |
|  | Tissue | .015 | .008 | .187 | 1.937 | .066 | -.001 | .031 |
|  | CBV | .036 | .004 | .873 | 9.249 | .000 | .028 | .044 |
|  | CBF | .000 | .000 | -.155 | -1.602 | .123 | -.001 | .000 |

| a. Dependent Variable: Ki |
| --- |

**8. ANCOVA of factors influencing K_i_**

| **Descriptive Statistics** | | | |
| --- | --- | --- | --- |
| Dependent Variable: Ki | | | |
| Tissue | Mean | Std. Deviation | N |
| WM | .00201619 | .038143743 | 13 |
| GM | .02681792 | .039906449 | 13 |
| Total | .01441706 | .040282978 | 26 |

| **Tests of Between-Subjects Effects** | | | | | |
| --- | --- | --- | --- | --- | --- |
| Dependent Variable: Ki | | | | | |
| Source | Type III Sum of Squares | df | Mean Square | F | Sig. |
| Corrected Model | .033^a^ | 3 | .011 | 32.450 | .000 |
| Intercept | .001 | 1 | .001 | 2.656 | .117 |
| CBF | .001 | 1 | .001 | 2.568 | .123 |
| CBV | .029 | 1 | .029 | 85.547 | .000 |
| Tissue | .001 | 1 | .001 | 3.751 | .066 |
| Error | .007 | 22 | .000 |  |  |
| Total | .046 | 26 |  |  |  |
| Corrected Total | .041 | 25 |  |  |  |

a. R Squared = .816 (Adjusted R Squared = .791)

**Estimated Marginal Means**

| **Tissue** | | | | |
| --- | --- | --- | --- | --- |
| Dependent Variable: Ki | | | | |
| Tissue | Mean | Std. Error | 95% Confidence Interval | |
|  |  |  | Lower Bound | Upper Bound |
| WM | .007^a^ | .005 | -.004 | .018 |
| GM | .022^a^ | .005 | .011 | .033 |

| a. Covariates appearing in the model are evaluated at the following values: CBF = 20.01166, CBV = .86606. |
| --- |

**9. Correlation of K_i_ and CBF (Spearman’s)**

| **Correlations** | | | | |
| --- | --- | --- | --- | --- |
|  | | | Ki | CBF |
| Spearman's rho | Ki | Correlation Coefficient | 1.000 | .320 |
|  |  | Sig. (2-tailed) | . | .111 |
|  |  | N | 26 | 26 |
|  | CBF | Correlation Coefficient | .320 | 1.000 |
|  |  | Sig. (2-tailed) | .111 | . |
|  |  | N | 26 | 26 |

**10. K_i_ values in control NAWM, RRMS NAWM, and CELs**

**Class = HC**

| **Descriptive Statistics^a^** | | | | | |
| --- | --- | --- | --- | --- | --- |
|  | N | Minimum | Maximum | Mean | Std. Deviation |
| WM_Ki_ROI | 13 | -.048765 | .114425 | .00201619 | .038143743 |
| Valid N (listwise) | 13 |  |  |  |  |

|  |
| --- |

**Class = RRMS**

| **Descriptive Statistics^a^** | | | | | |
| --- | --- | --- | --- | --- | --- |
|  | N | Minimum | Maximum | Mean | Std. Deviation |
| WM_Ki_ROI | 12 | -.007862 | .116360 | .05235622 | .037319941 |
| Valid N (listwise) | 12 |  |  |  |  |

|  |
| --- |

**Class = CEL**

| **Descriptive Statistics^a^** | | | | | |
| --- | --- | --- | --- | --- | --- |
|  | N | Minimum | Maximum | Mean | Std. Deviation |
| WM_Ki_ROI | 4 | .071200 | .164400 | .13975000 | .045725011 |
| Valid N (listwise) | 4 |  |  |  |  |

|  |
| --- |

**11. Comparison of Ki in control NAWM and CELs (Mann-Whitney)**

|  |
| --- |

**Mann-Whitney Test**

| **Ranks** | | | | |
| --- | --- | --- | --- | --- |
|  | Class | N | Mean Rank | Sum of Ranks |
| WM_Ki_ROI | HC | 13 | 7.08 | 92.00 |
|  | CEL | 4 | 15.25 | 61.00 |
|  | Total | 17 |  |  |

| **Test Statistics^a^** | |
| --- | --- |
|  | WM_Ki_ROI |
| Mann-Whitney U | 1.000 |
| Wilcoxon W | 92.000 |
| Z | -2.831 |
| Asymp. Sig. (2-tailed) | .005 |
| Exact Sig. [2*(1-tailed Sig.)] | .002^b^ |

| a. Grouping Variable: Class |
| --- |
| b. Not corrected for ties. |

**12. Comparison of K_i_ in RRMS NAWM and CELs (t-test)**

| **Group Statistics** | | | | | |
| --- | --- | --- | --- | --- | --- |
|  | Class | N | Mean | Std. Deviation | Std. Error Mean |
| WM_Ki_ROI | RRMS | 12 | .05235622 | .037319941 | .010773339 |
|  | CEL | 4 | .13975000 | .045725011 | .022862506 |

| **Independent Samples Test** | | | | | | |
| --- | --- | --- | --- | --- | --- | --- |
|  | | Levene's Test for Equality of Variances | | t-test for Equality of Means | | |
|  |  | F | Sig. | t | df | Sig. (2-tailed) |
|  |  |  |  |  |  |  |
| WM_Ki_ROI | Equal variances assumed | .218 | .648 | -3.854 | 14 | .002 |
|  | Equal variances not assumed |  |  | -3.458 | 4.421 | .022 |

| **Independent Samples Test** | | | | | |
| --- | --- | --- | --- | --- | --- |
|  | | t-test for Equality of Means | | | |
|  |  | Mean Difference | Std. Error Difference | 95% Confidence Interval of the Difference | |
|  |  |  |  | Lower | Upper |
| WM_Ki_ROI | Equal variances assumed | -.087393784 | .022674135 | -.136024966 | -.038762602 |
|  | Equal variances not assumed | -.087393784 | .025273682 | -.155007580 | -.019779987 |

**13. Comparison of NAWM K_i_ by ROI method between controls and RRMS (ANCOVA)**

| **Between-Subjects Factors** | | | |
| --- | --- | --- | --- |
|  | | Value Label | N |
| Class | .00 | HC | 13 |
|  | 1.00 | RRMS | 12 |

| **Descriptive Statistics** | | | |
| --- | --- | --- | --- |
| Dependent Variable: WM_Ki_ROI | | | |
| Class | Mean | Std. Deviation | N |
| HC | .00201619 | .038143743 | 13 |
| RRMS | .05235622 | .037319941 | 12 |
| Total | .02617940 | .044996666 | 25 |

| **Tests of Between-Subjects Effects** | | | | | | |
| --- | --- | --- | --- | --- | --- | --- |
| Dependent Variable: WM_Ki_ROI | | | | | | |
| Source | Type III Sum of Squares | df | Mean Square | F | Sig. | Partial Eta Squared |
| Corrected Model | .016^a^ | 2 | .008 | 5.361 | .013 | .328 |
| Intercept | .001 | 1 | .001 | .435 | .516 | .019 |
| Age | .000 | 1 | .000 | .073 | .789 | .003 |
| Class | .011 | 1 | .011 | 7.189 | .014 | .246 |
| Error | .033 | 22 | .001 |  |  |  |
| Total | .066 | 25 |  |  |  |  |
| Corrected Total | .049 | 24 |  |  |  |  |

| a. R Squared = .328 (Adjusted R Squared = .267) |
| --- |

**Estimated Marginal Means**

| **Class** | | | | |
| --- | --- | --- | --- | --- |
| Dependent Variable: WM_Ki_ROI | | | | |
| Class | Mean | Std. Error | 95% Confidence Interval | |
|  |  |  | Lower Bound | Upper Bound |
| HC | .003^a^ | .012 | -.021 | .027 |
| RRMS | .051^a^ | .012 | .026 | .076 |

| a. Covariates appearing in the model are evaluated at the following values: Age = 36.68. |
| --- |

**14. Comparison of NAWM Ki by segmentation method between controls and RRMS (ANCOVA)**

| **Between-Subjects Factors** | | | |
| --- | --- | --- | --- |
|  | | Value Label | N |
| Class | .00 | HC | 13 |
|  | 1.00 | RRMS | 12 |

| **Descriptive Statistics** | | | |
| --- | --- | --- | --- |
| Dependent Variable: WM_Ki_SEG | | | |
| Class | Mean | Std. Deviation | N |
| HC | .00292308 | .027288818 | 13 |
| RRMS | .05937525 | .045472086 | 12 |
| Total | .03002012 | .046353219 | 25 |

| **Tests of Between-Subjects Effects** | | | | | | |
| --- | --- | --- | --- | --- | --- | --- |
| Dependent Variable: WM_Ki_SEG | | | | | | |
| Source | Type III Sum of Squares | df | Mean Square | F | Sig. | Partial Eta Squared |
| Corrected Model | .024^a^ | 2 | .012 | 9.461 | .001 | .462 |
| Intercept | .000 | 1 | .000 | .319 | .578 | .014 |
| Age | .004 | 1 | .004 | 3.141 | .090 | .125 |
| Class | .008 | 1 | .008 | 6.435 | .019 | .226 |
| Error | .028 | 22 | .001 |  |  |  |
| Total | .074 | 25 |  |  |  |  |
| Corrected Total | .052 | 24 |  |  |  |  |

| a. R Squared = .462 (Adjusted R Squared = .414) |
| --- |

**Estimated Marginal Means**

| **Class** | | | | |
| --- | --- | --- | --- | --- |
| Dependent Variable: WM_Ki_SEG | | | | |
| Class | Mean | Std. Error | 95% Confidence Interval | |
|  |  |  | Lower Bound | Upper Bound |
| HC | .010^a^ | .011 | -.012 | .032 |
| RRMS | .052^a^ | .011 | .029 | .075 |

| a. Covariates appearing in the model are evaluated at the following values: Age = 36.68. |
| --- |

**15. Test for non-zero mean difference between methods (t-test)**

| **One-Sample Statistics** | | | | |
| --- | --- | --- | --- | --- |
|  | N | Mean | Std. Deviation | Std. Error Mean |
| Diff | 25 | -.00384072 | .040328593 | .008065719 |

| **One-Sample Test** | | | | | | |
| --- | --- | --- | --- | --- | --- | --- |
|  | Test Value = 0 | | | | | |
|  | t | df | Sig. (2-tailed) | Mean Difference | 95% Confidence Interval of the Difference | |
|  |  |  |  |  | Lower | Upper |
| Diff | -.476 | 24 | .638 | -.003840719 | -.02048754 | .01280611 |

**16. Bland-Altman plot**


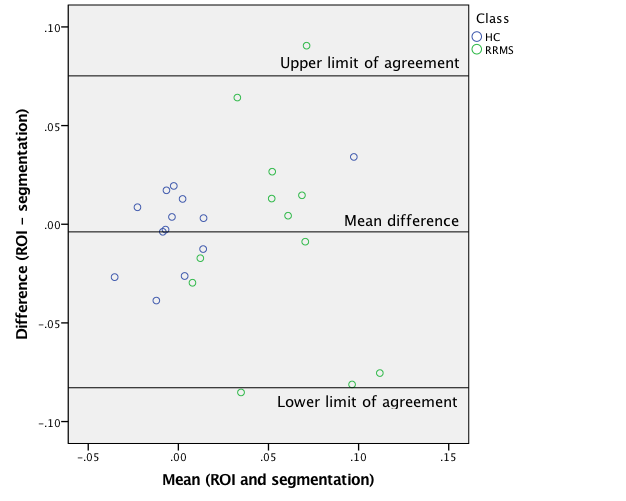


**17. Regression for proportional bias**

| **Variables Entered/Removed^a^** | | | |
| --- | --- | --- | --- |
| Model | Variables Entered | Variables Removed | Method |
| 1 | Mean^b^ | . | Enter |

| a. Dependent Variable: Diff |
| --- |
| b. All requested variables entered. |

| **Model Summary** | | | | |
| --- | --- | --- | --- | --- |
| Model | R | R Square | Adjusted R Square | Std. Error of the Estimate |
| 1 | .037^a^ | .001 | -.042 | .041167023 |

| a. Predictors: (Constant), Mean |
| --- |

| **ANOVA^a^** | | | | | | |
| --- | --- | --- | --- | --- | --- | --- |
| Model | | Sum of Squares | df | Mean Square | F | Sig. |
| 1 | Regression | .000 | 1 | .000 | .032 | .859^b^ |
|  | Residual | .039 | 23 | .002 |  |  |
|  | Total | .039 | 24 |  |  |  |

| a. Dependent Variable: Diff |
| --- |
| b. Predictors: (Constant), Mean |

| **Coefficients^a^** | | | | | | |
| --- | --- | --- | --- | --- | --- | --- |
| Model | | Unstandardized Coefficients | | Standardized Coefficients | t | Sig. |
|  |  | B | Std. Error | Beta |  |  |
| 1 | (Constant) | -.003 | .010 |  | -.279 | .783 |
|  | Mean | -.037 | .205 | -.037 | -.180 | .859 |

| a. Dependent Variable: Diff |
| --- |

**18. Intraclass correlation coefficient**

| **Case Processing Summary** | | | |
| --- | --- | --- | --- |
|  | | N | % |
| Cases | Valid | 25 | 100.0 |
|  | Excluded^a^ | 0 | .0 |
|  | Total | 25 | 100.0 |

| a. Listwise deletion based on all variables in the procedure. |
| --- |

| **Reliability Statistics** | |
| --- | --- |
| Cronbach's Alpha | N of Items |
| .758 | 2 |

| **Intraclass Correlation Coefficient** | | | | | | | | |
| --- | --- | --- | --- | --- | --- | --- | --- | --- |
|  | Intraclass Correlation^b^ | 95% Confidence Interval | | F Test with True Value 0 | | | |  |
|  |  | Lower Bound | Upper Bound | Value | df1 | df2 | Sig |  |
| Single Measures | .610^a^ | .291 | .807 | 4.132 | 24 | 24 | .000 |  |
| Average Measures | .758^c^ | .451 | .893 | 4.132 | 24 | 24 | .000 |  |

| Two-way mixed effects model where people effects are random and measures effects are fixed. |
| --- |
| a. The estimator is the same, whether the interaction effect is present or not. |
| b. Type C intraclass correlation coefficients using a consistency definition. The between-measure variance is excluded from the denominator variance. |
| c. This estimate is computed assuming the interaction effect is absent, because it is not estimable otherwise. |
